# Nudges and Prompts Increase Engagement in Self-Guided Digital Health Treatment for Depression and Anxiety: Results From a 3-Arm Randomized Controlled Trial

**DOI:** 10.2196/52558

**Published:** 2024-04-09

**Authors:** Trevor van Mierlo, Renante Rondina, Rachel Fournier

**Affiliations:** 1 Evolution Health Toronto, ON Canada; 2 Rotman School of Managment University of Toronto Toronto, ON Canada

**Keywords:** behavioral economics, digital health, attrition, engagement, nudges, depression, anxiety, mood disorders

## Abstract

**Background:**

Accessible and effective approaches to mental health treatment are important because of common barriers such as cost, stigma, and provider shortage. The effectiveness of self-guided treatment is well established, and its use has intensified because of the COVID-19 pandemic. Engagement remains important as dose-response relationships have been observed. Platforms such as Facebook (Meta Platform, Inc), LinkedIn (Microsoft Corp), and X Corp (formerly known as Twitter, Inc) use principles of behavioral economics to increase engagement. We hypothesized that similar concepts would increase engagement in self-guided digital health.

**Objective:**

This 3-arm randomized controlled trial aimed to test whether members of 2 digital self-health courses for anxiety and depression would engage with behavioral nudges and prompts. Our primary hypothesis was that members would click on 2 features: tips and a to-do checklist. Our secondary hypothesis was that members would prefer to engage with directive tips in arm 2 versus social proof and present bias tips in arm 3. Our tertiary hypothesis was that rotating tips and a to-do checklist would increase completion rates. The results of this study will form a baseline for future artificial intelligence–directed research.

**Methods:**

Overall, 13,224 new members registered between November 2021 and May 2022 for Evolution Health’s self-guided treatment courses for anxiety and depression. The control arm featured a member home page without nudges or prompts. Arm 2 featured a home page with a tip-of-the-day section. Arm 3 featured a home page with a tip-of-the-day section and a to-do checklist. The research protocol for this study was published in *JMIR Research Protocols* on August 15, 2022.

**Results:**

Arm 3 had significantly younger members (*F*_2,4564_=40.97; *P*<.001) and significantly more female members (*χ*^2^_4_=92.2; *P*<.001) than the other 2 arms. Control arm members (1788/13,224, 13.52%) completed an average of 1.5 course components. Arm 2 members (865/13,224, 6.54%) clicked on 5% of tips and completed an average of 1.8 course components. Arm 3 members (1914/13,224, 14.47%) clicked on 5% of tips, completed 2.7 of 8 to-do checklist items, and completed an average of 2.11 course components. Completion rates in arm 2 were greater than those in arm 1 (*z* score=3.37; *P*<.001), and completion rates in arm 3 were greater than those in arm 1 (*z* score=12.23; *P*<.001). Engagement in all 8 components in arm 3 was higher than that in arm 2 (*z* score=1.31; *P*<.001).

**Conclusions:**

Members engaged with behavioral nudges and prompts. The results of this study may be important because efficacy is related to increased engagement. Due to its novel approach, the outcomes of this study should be interpreted with caution and used as a guideline for future research in this nascent field.

**International Registered Report Identifier (IRRID):**

RR2-10.2196/37231

## Introduction

### Background

Since its earliest use in the mid-1990s, digital health promised personalized treatments that patients could access from home. It was anticipated that treatment would have a broad reach, resulting in improved health outcomes and decreased costs [1-3]. Over the past 2 decades, and with increasing consistency, research examining the efficacy of self-guided digital health interventions show evidence of efficacy, especially for those with mental health concerns [4-7].

Although digital health interventions appear to be effective, poor adherence and lack of compliance have remained consistent patterns in research [8-10]. This pattern was first recognized in 2005 and coined *The Law of Attrition* [11].

As early as 2009, systematic reviews identified poor adherence and lack of compliance as engagement issues that required attention [12]. These issues persist, as demonstrated by a recent meta-analytic review on digital interventions for depression, which showed efficacy but highlighted compliance as a major challenge [13].

Adherence and compliance are complex and rooted in several systemic and individual factors [14-19]. However, it is an important topic as evidence indicates a dose-response relationship, and higher levels of engagement are associated with improved health outcomes [20,21].

Moreover, digital health interventions are becoming increasingly common and accessible. Patients’ use of, and trust, in these interventions has been intensified by the COVID-19 pandemic. The use of self-guided digital health interventions for mental health concerns is growing [22,23] because of access barriers such as high cost, stigma, and lack of access due to a shortage of professionals who can meet this growing demand [24,25].

How do we increase engagement in digital health programs to maximize their efficacy?

### Behavioral Economics

#### Overview

Behavioral economics leverages psychological experimentation to develop theories about human decision-making. The field has identified a range of unconscious biases around how people think and feel [26,27].

The utility of behavioral economics is vast. Digital health has leveraged the discipline to investigate how people use digital health programs and to gain insights into the characteristics of people who use them. Several digital health studies have investigated the use of these strategies, including cooperative games and incentives [28], gamification [29,30], *serious games* [31,32], and positive behavioral support [33,34].

#### Our Use of Behavioral Economics

In our study, we examined the effectiveness of the nudge theory and behavioral prompts in 2 ad libitum self-guided digital behavior change courses.

#### Nudge Theory

Nudge theory, popularized in the 2008 book *Nudge: Improving Decisions About Health, Wealth, and Happiness* [27]*,* leverages indirect, positive suggestions to influence decision-making and behavior.

There is a lack of quality research analyzing the use of nudges in digital health. A 2019 scoping review examined the use of nudges in both web-based and real-world settings in physical activity interventions [35]. Of the 35 publications reviewed, 8 were web-based studies. The authors concluded that although nudging may be an effective approach to promote physical activity, there are large gaps in research, and further studies that are explicitly based on nudge insights are needed.

A 2020 editorial in *Personalized Medicine* addressed the meaningful adoption of nudges in digital health [36]. The authors acknowledged that using nudges in digital health interventions is rare and advocated for the use of nudges to promote positive behavior change.

#### Behavioral Prompts

In applied behavioral analysis, behavioral prompts are cues specifically designed to encourage a specific task [34]. In this study, we used 2 types of behavioral prompts anchored in the nudge theory: daily tips and a to-do checklist (Table 1).

**Table 1:** Example nudges and prompts.

Delivery format	Content type	Example text from our study
Tip	Directive content	Express yourself by uploading your image
Tip	Social proof	Many members have similar goals as you. Reviewing other members’ goals can help you reach your goal.
Tip	Present bias	Feel better sooner by learning from others. Read what others have posted on the community.
To-do checklist	Directive content	Watch the getting started video

#### Directive, Social Proof, and Present Bias Tips

Directive content, as the name suggests, offers concrete suggestions to members [37]. These types of tips are brief and instructional.

Social proof is derived from the behavioral economics concept where we tend to copy the actions of those around us. These tips speak of our tendency to be swayed by other people’s choices, which we attempt to mirror [38,39].

Present bias is the inclination to prefer a smaller present reward now over a larger reward later. These tips encourage users to perform a task that provides an immediate benefit [40,41].

#### Our Use of Behavioral Prompts and Technical Functionality of Tips and to-Do Checklist Items

An example of a course tool in both Overcoming Anxiety and Overcoming Depression courses is goal setting. Goal setting is an important component of cognitive behavioral therapy (CBT). However, in a self-guided environment, many members are unsure of how to set personal goals. Evolution Health encourages members to review goals set by others as examples.

**Figure 1:**
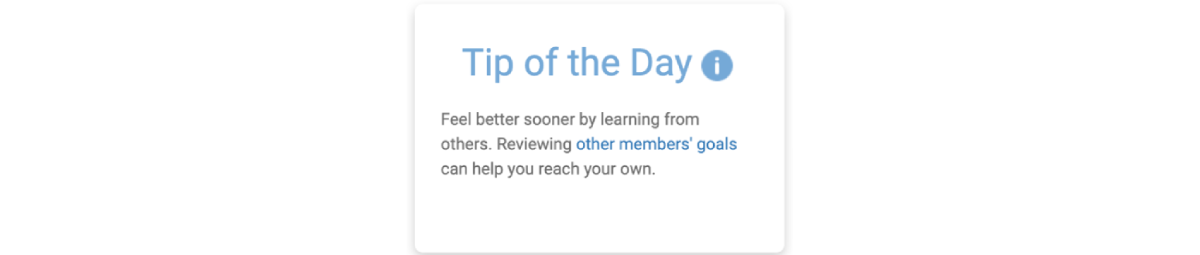
Present bias tip: review other members’ goals.

Figure 1 shows an example of a tip of the day that encourages members to review the goals of other members.

**Figure 2:**
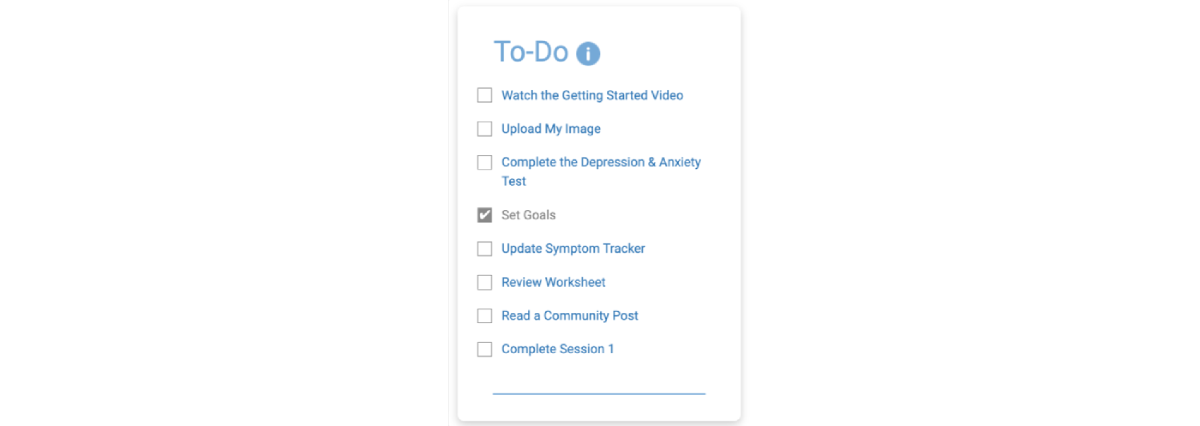
Completed to-do checklist item, set goals.

Figure 2 shows an example of the completed to-do checklist item, *set goals*. In this figure, the item is marked complete by a check box. This signifies that a member has clicked on the item and visited the page.

**Figure 3:**
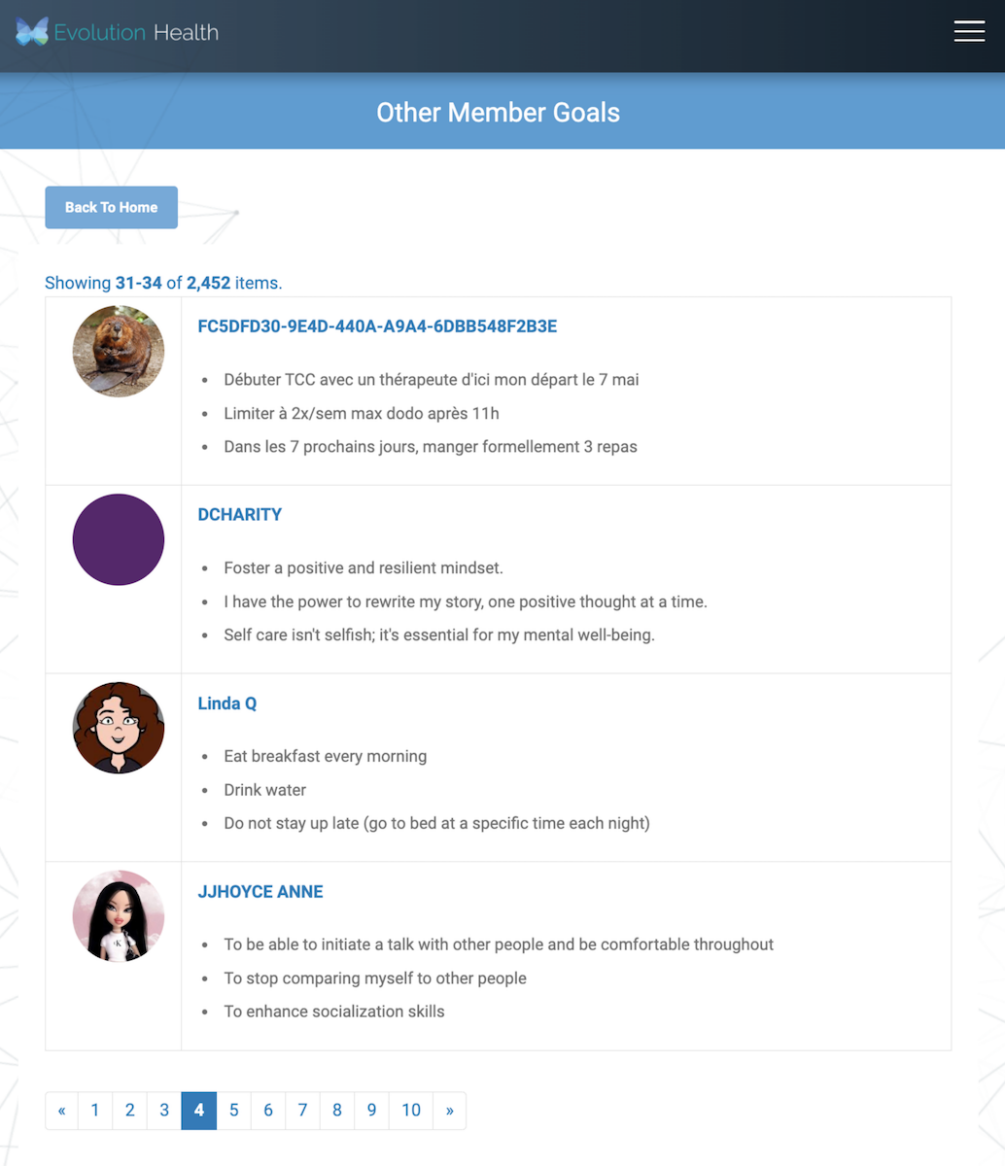
Other members’ goals.

By clicking on either the tip (Figure 2) or the to-do checklist item (Figure 3), the member is brought to member goals, a course component that allows members to browse various goals set by other members (Figure 3).

Engagement experiments in popular non–health care digital platforms are common. Although they are scientific in nature, they are not typically published, as they are conducted within private companies.

For example, social network sites such as Facebook (Meta Platforms, Inc), LinkedIn (Microsoft Corp), and X Corp. (formerly known as Twitter) generate revenue based on ad revenue derived from page views and the time members spend on their site. In a 2015 presentation, it was revealed that LinkedIn had >400 controlled experiments being conducted per day [42]. Similar studies with an ad libitum population are required for digital health, and this study is an attempt to fill this gap.

We have not observed sufficient evidence in the literature to determine whether nudges and prompts can be strategically applied to increase engagement with and decrease attrition in the courses for depression and anxiety [43]. Furthermore, because of the nascent state of behavioral economics within digital health, we did not find any quantitative benchmarks that would help us determine whether our use of nudges and prompts was successful.

### Objectives

Our primary hypothesis (H1) was that members would engage with the tips and to-do checklist. Engagement of the members was determined by the percentage of tips that were clicked on compared to the number shown. Because there is no prior literature, we first needed to establish baseline data. Data were reported as observational and served as a benchmark for future studies.

Our secondary hypothesis was that members would prefer to engage with directive tips in arm 2 versus social proof or present bias tips in arm 3. To assess the preference for engagement, we compared the number of tips clicked in arm 2 versus those clicked in arm 3.

Our tertiary hypothesis was that the addition of tips and a to-do checklist would increase completion rates with course tools. Increased completion rates were determined by comparing completion rates in arms 2 and 3 with those in arm 1 (control group).

In each hypothesis, we assessed whether engagement was influenced by gender or age.

## Methods

### Overview

The digital health platform used in this study was managed by Evolution Health. Evolution Health is an evidence-based, self-guided digital health platform that features courses and brief interventions based on behavior change techniques including CBT, stages of change, structured relapse prevention, normative feedback, and harm reduction.

The platform offers interactive courses and quizzes for people with mental health, addiction, and obesity issues. The platform contains a moderated community based on social cognitive theory.

Memberships are available to individuals who register through the organization’s free-to-consumer program and white-label instances that are licensed by employers, insurance companies, employee assistance programs, educational institutions, nonprofit organizations, for-profit health care organizations, and individual therapists.

The research protocol for this study was published in *JMIR Research Protocols* on August 15, 2022 [44]. The International Registered Report Identifier is DERR1-10.2196/37231.

### The Interventions

The 2 interventions in this study contain self-guided interactive behavior change treatment courses based on best practices, and both have been examined extensively in the literature [9,45-53].

The 2 interventions have undergone several iterations. For example, Overcoming Anxiety was the first intervention noted in *The* *Law of Attrition* (previously known as The Panic Center) paper by Eysenbach [11]. In that iteration, the course contained a tunnel design with 12 successive sessions. The course now has a gamified free-form matrix design, among other technical and usability enhancements.

Table 2 outlines each course’s current theoretical construct and evidence base. Table 3 outlines the main course components.

**Table 2:** Theoretical constructs and evidence base.

Theoretical construct	Overcoming Depression course	Overcoming Anxiety course
Brief intervention	✓	✓
Cognitive behavioral therapy	✓	✓
Gamification	✓	✓
Health belief model	✓	✓
Motivational interviewing	✓	✓
Normative feedback	✓	✓
Social cognitive theory	✓	✓
Targeting and tailoring	✓	✓

**Table 3:** Main course components.

Course component	Overcoming Depression course	Overcoming Anxiety course
Avatar upload	✓	✓
Course completion certificate	✓	✓
Course diary (mood tracker or symptom tracker)	✓	✓
Course worksheets	✓	✓
Gamified CBT^a^ course	✓	✓
Getting started video	✓	✓
Goals exercise	✓	✓
Moderated community	✓	✓
Private messaging	✓	✓
Statistics interface (for corporate clients)	✓	✓
Tailored depression and anxiety test	✓	✓
Therapist interface	✓	✓

^a^CBT: cognitive behavioral therapy.

### Ethical Considerations

At registration, all members endorsed a checkbox to confirm that they consented to have their nonidentifiable data used for research purposes and approved the platform’s privacy policy. The participants did not receive compensation for their involvement. The platform is available in several languages, and the English language privacy policy and terms of use are presented in Multimedia Appendix 1. All data collection policies and procedures adhered to the international privacy guidelines [54-56] and the Helsinki Declaration of 1975 [57].

This study was conducted on self-guided treatment for depression and anxiety, but it did not measure clinical outcomes. Although the study participants were randomly assigned to a control or intervention arm, the study was a randomized controlled trial and not a randomized clinical trial [58]. This study did not assess whether the study participants’ engagement with course tools decreased the depressive symptoms, severity of panic attacks, or frequency of panic attacks.

As described earlier, evidence indicates that higher levels of engagement are associated with improved health outcomes, and the literature observes dose-response relationships. However, any clinical outcomes related to the engagement strategies used in this study will need to be tested in future research.

As the study was based on unidentifiable data and no clinical measures were tested, it was deemed exempt from further review by the Evolution Health Institutional Review Board (IRB#000014034, FWA00033737).

### Power and Sample Size

The study was designed to have a power of 0.95, indicating a 95% probability of correctly detecting a statistically significant difference in engagement with tips between the 2 treatment groups (arm 2 vs arm 3). On the basis of a 2-tailed *t* test with a conventional α level (.05) for statistical significance, we required a sample of 1302 members with 651 in each group to detect a small effect size of Cohen *d*=0.2. Refer to Multimedia Appendix 2 for the CONSORT-EHEALTH (Consolidated Standards of Reporting Trials of Electronic and Mobile Health Applications and Online Telehealth) checklist [59].

### Randomization

**Figure 4:**
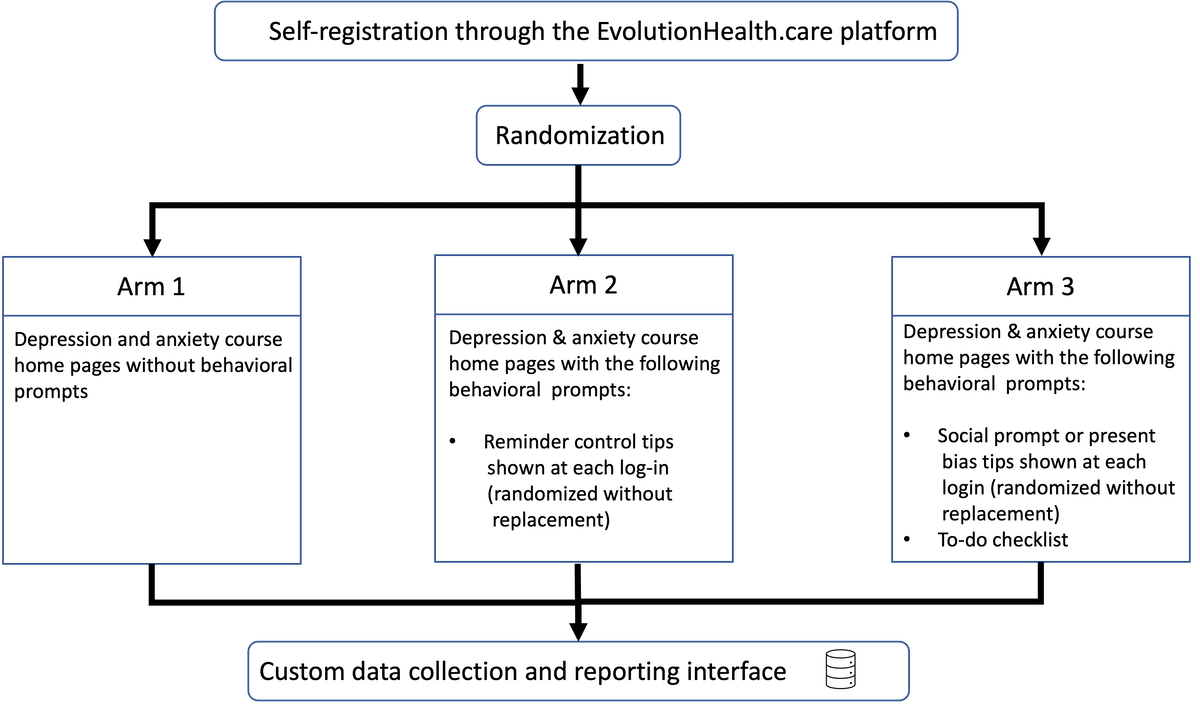
Recruitment process.

During the registration process, new members were assigned to 1 of the 3 arms using a random number generator (Figure 4). Randomization was conducted using simple randomization.

### Intervention Groups

#### Arm 1

**Figure 5:**
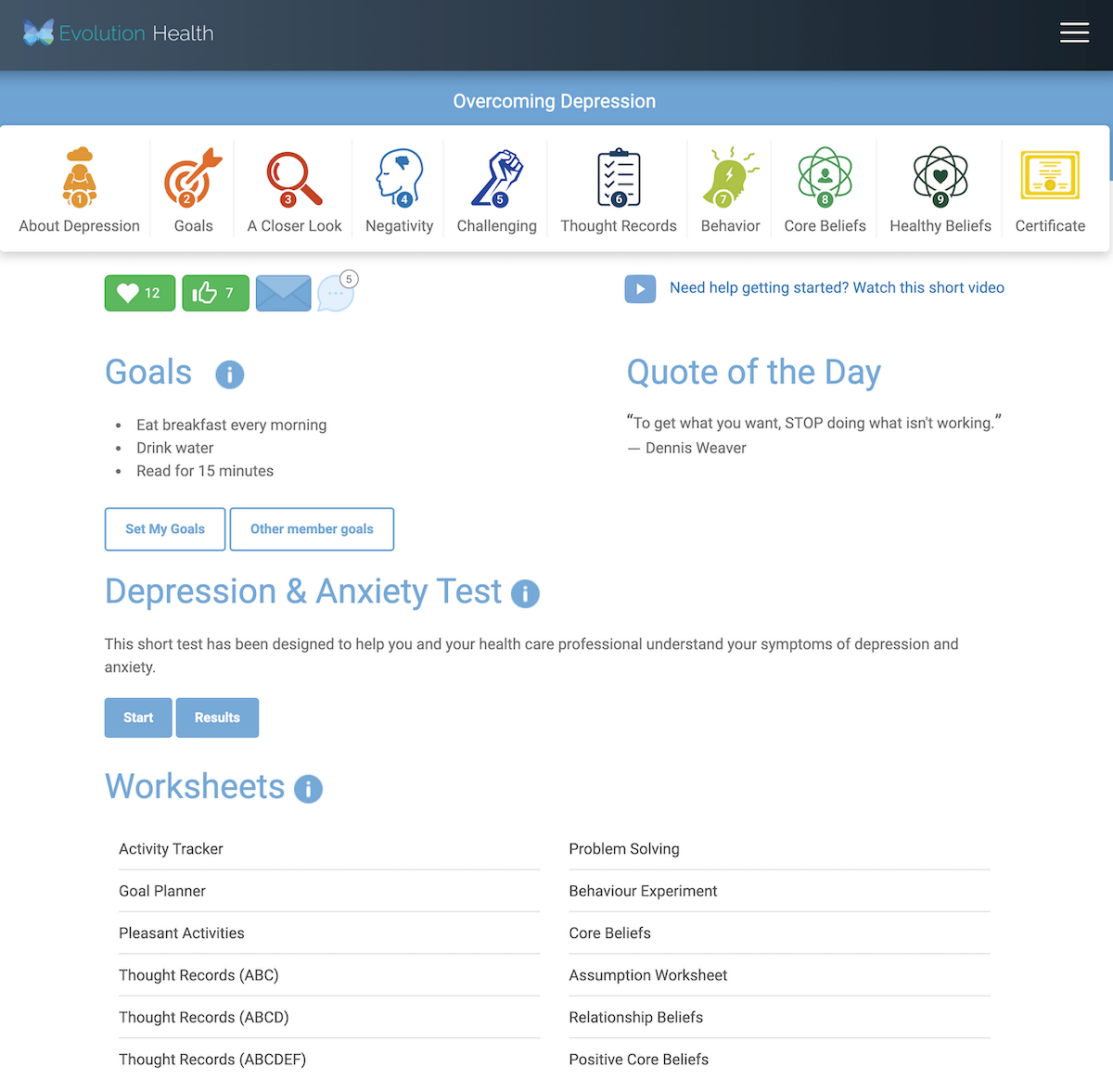
Member home page for arm 1.

Members randomized to arm 1 were presented with a dashboard that did not contain behavioral nudges. Figure 5 presents a screenshot of the arm 1 dashboard for a member who chose to engage with the depression course.

#### Arm 2

Members randomized to arm 2 were presented with a dashboard that contained a tip-of-the-day section containing directive content. The randomization strategy for the 31 directive tips was randomization without replacement.

**Figure 6:**
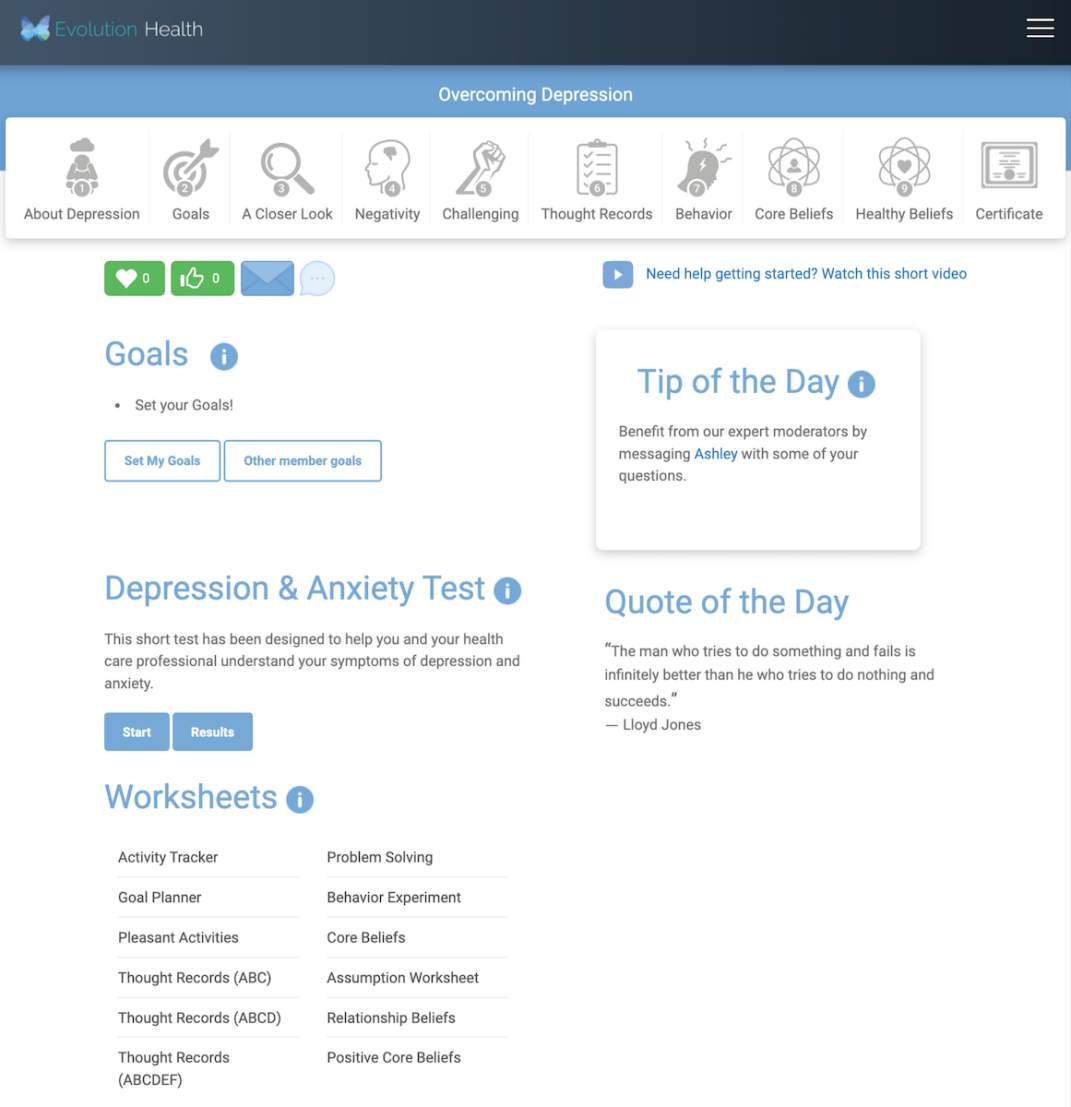
Member home page for arm 2.

Figure 6 presents a screenshot of the arm 2 dashboard for a member who chose to engage with the depression course.

#### Arm 3

Members randomized to arm 3 were presented with 2 sections that contained nudges. The first was a tip-of-the-day section containing social proof and present bias cues. At each log-in, members saw a new tip. The randomization strategy for the tips was randomization without replacement. There were 15 social proof tips and 15 present bias tips.

In addition to the tip of the day, arm 3 featured a to-do checklist that listed 8 course components. When a member clicked on a component, they were brought to the exercise. As mentioned previously, if a member clicks on a to-do checklist item, it is marked complete with a check mark.

**Figure 7:**
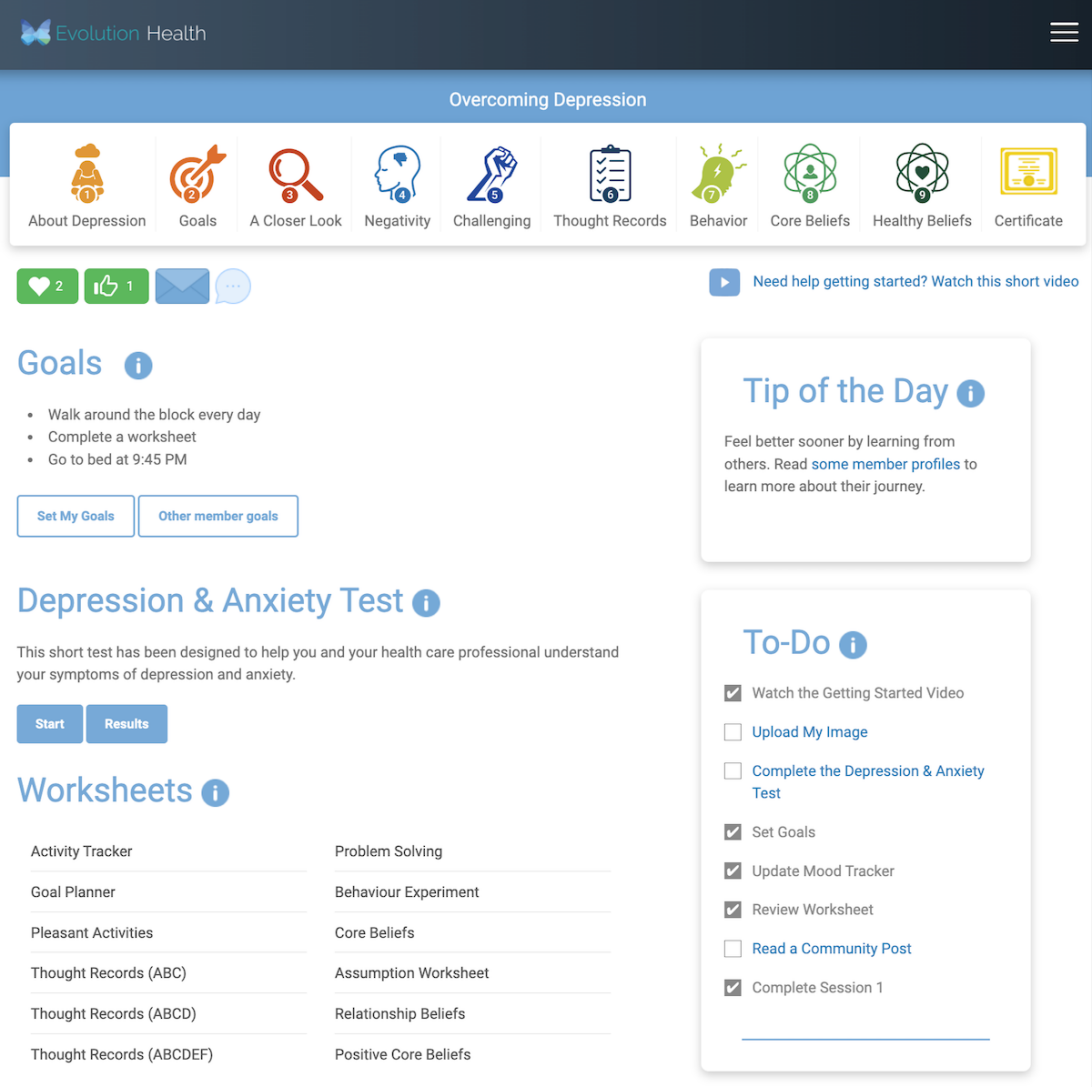
Member home page for arm 3.

Figure 7 presents a screenshot of the arm 2 dashboard for a member who chose to engage with the depression course.

### Data Collection

A custom data collection interface and reporting mechanism were developed by Evolution Health. Age and gender data were collected at registration or through a secure sign on with various white-label instances. Data were collected for each member who was randomized into the experiment. The course components promoted by tips and to-do checklist items are listed in Textbox 1. Members who participated in both courses were not counted twice.

The following behaviors were tracked in the custom database for each tip and to-do checklist item that was randomly presented to a member:

- If the nudge was shown
- If the nudge was clicked on
- If a member completed the course component described in the tip or to-do checklist item.

It was possible for a single member to participate in both courses. However, the study design was to test the behavioral prompts, not the courses. The participants were randomized to an intervention arm, in which behavioral nudges and prompts were consistent across courses.

**Course components tracked for engagement.**

**Action code and course component**

- 1 (item in the to-do checklist): Uploading a personal image (avatar) to their profile
- 2 (item in the to-do checklist): Completing cognitive behavioral therapy session 1
- 3 (item in the to-do checklist): Use of the program diary (mood tracker or symptom tracker)
- 4 (item in the to-do checklist): Read a community post
- 5 (item in the to-do checklist): Review a worksheet
- 6 (item in the to-do checklist): Set personal goals
- 7 (item in the to-do checklist): Complete the depression and anxiety test
- 8 (item in the to-do checklist): Watch the getting started video
- 9: Review another member’s profile
- 10: Post in the community
- 11: Read other member’s goals
- 12: Give a community member a “thumbs up”
- 13: Encourage a community member by clicking their “show support” icon
- 14: Private message a community moderator

### Participants

As there were no barriers to registration and many new members registered with the platform for purposes other than treatment, we removed those who registered but did not return to the intervention (nonparticipants) from the analysis.

### Data Analyses

All data were analyzed using mixed effect logistic regression with members as a random variable. Mixed effect logistic regression was conducted using the *glmer()* function from the *lme4* package in R software (R Foundation for Statistical Computing), with the default optimizer Bound Optimization BY Quadratic Approximation, a derivative-free optimization algorithm used for problems with bound constraints.

For the tip-of-the-day section, the outcome variable was whether a user clicked on a tip presented to them (no or yes), and the predictors were arm (2 or 3), gender (female or male), and age group (18 to 30 years to >60 years).

For the to-do checklist items, the outcome variable was whether a user clicked on a checklist item (no or yes), and the predictors were gender and age group.

For the course components, the outcome variable was whether a user completed a component (no or yes), and the predictors were arm (1, 2, or 3), gender, and age group.

## Results

### Overview

Between November 2021 and May 2022, data were collected from new members who self-registered for Evolution Health’s self-guided treatment program for anxiety and depression. All members were randomized into 1 of the 3 arms.

First, members with test accounts and unauthenticated accounts were removed from the data set, resulting in a population of 13,224 members. Then, members whose accounts were missing demographic data (376/13,224, 2.84%) were removed from the data set, followed by members aged <18 (129/13,224, 0.97%), resulting in a population of 12,719 members.

**Figure 8:**
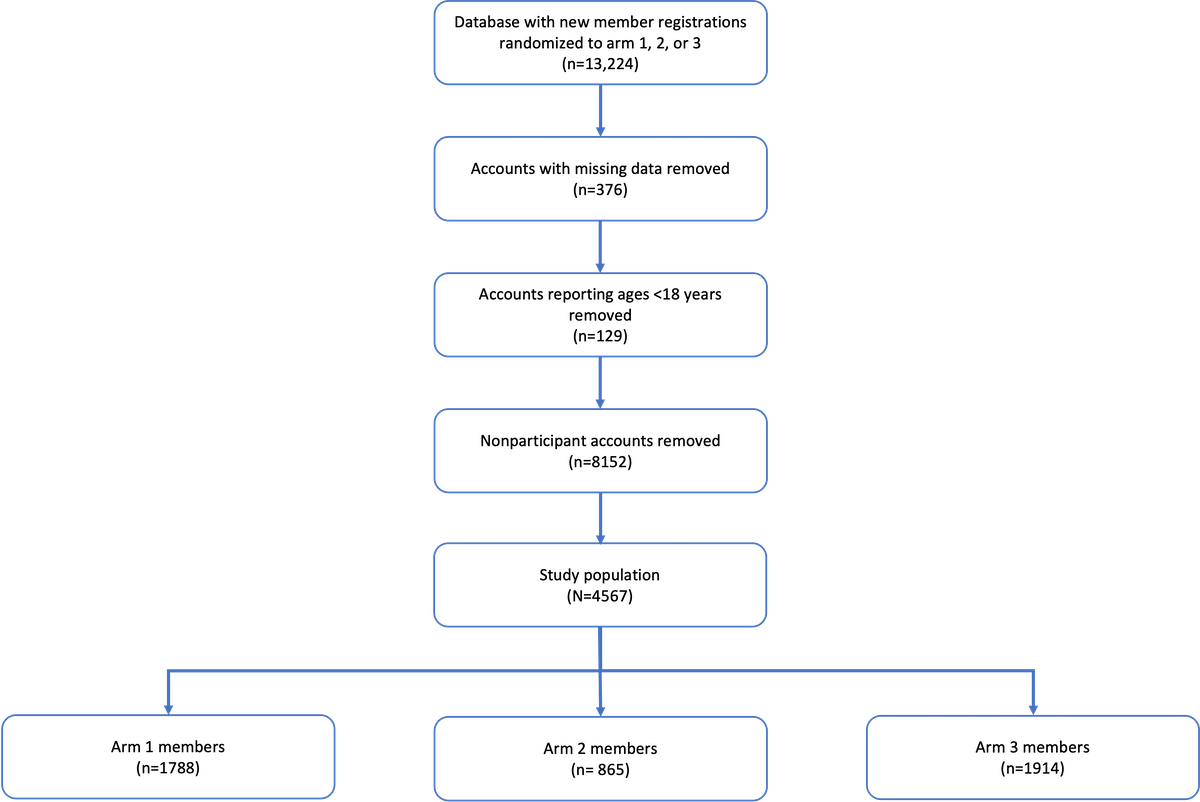
Study population.

Finally, of the 13,224 members, 8567 (65.46%) with nonparticipant accounts were removed, resulting in a study population of 4567 (34.53%). Of the 4567 members, 1788 (39.15%) were randomized into arm 1, 865 (18.94%) into arm 2, and 1914 (41.9%) into arm 3 (Figure 8).

### Demographic Characteristics

The average age of the members in arms 1, 2, and 3 was 44.4 (SD 10.69), 45.4 (SD 10.62), and 41.8 (SD 11.17), respectively (Table 4). The 51 to 60 years age group was the most populous age category in each of the arms (arm 1: 777/1788, 43.45%; arm 2: 420/865, 48.6%; and arm 3: 586/1914, 30.61%). The members of arm 3 were somewhat younger (*F*_2,4564_=40.97; *P*<.001) than the other 2 arms.

Regarding gender (Table 5), most members identified as women (arm 1: 1466/1788, 81.99%; arm 2: 751/865, 86.8%; and arm 3: 1770/1914, 92.48%). Few members identified as transgender individuals, nonbinary or third gender individuals, or preferred not to say (arm 1: 5/1788, 0.28%; arm 2: 1/865, 1.2%; and arm 3: 2/1914, 0.1%). Arm 3 had more female members (*χ*^2^_4_=92.21; *P*<.001) than the other 2 arms.

During the study period, there were 13,510 total log-ins, with initial registration considered as 1 log-in (Table 6). The average number of log-ins for male members was 3.89 and female members was 2.83. The average visit duration (AVD) for members who engaged with depression and anxiety course tools ranged from 6 minutes and 45 seconds, with 7.65 pages viewed, to 24 minutes and 21 seconds, with 12.56 pages viewed.

**Table 4:** Age breakdown per arm (N=4567).

Age range (years)	Arm 1 (n=1788), n (%)	Arm 2 (n=865), n (%)	Arm 3 (n=1914), n (%)	Total age category, n (%)
18 to 30	231 (12.91)	108 (12.5)	368 (19.22)	707 (15.48)
31 to 40	393 (21.97)	159 (18.4)	492 (25.7)	1044 (22.86)
41 to 50	329 (18.4)	147 (17.0)	415 (21.68)	891 (19.51)
51 to 60	777 (43.45)	420 (48.6)	586 (30.61)	1783 (39.04)
>60	58 (3.24)	32 (3.7)	53 (2.76)	142 (3.11)

**Table 5:** Gender categories per arm (N=4567).

Gender	Arm 1 (n=1788), n (%)	Arm 2 (n=856), n (%)	Arm 3 (n=1914), n (%)	Total, n (%)
Female members	1466 (81.99)	751 (86.8)	1770 (92.48)	3987 (87.3)
Male members	317 (17.72)	113 (13.1)	142 (7.42)	572 (12.52)
Other gender identity	5 (0.28)	1 (1.2)	2 (0.1)	8 (0.17)

**Table 6:** Number of log-ins by gender category.

Gender	Maximum number of log-ins, n	Minimum number of log-ins, n	Average number of log-ins, mean (SD)
Female members	276	1	2.83 (6.04)
Male members	243	1	3.89 (12.13)
Other gender identity	6	1	2.6 (1.85)
Range	276	1	2.38 (7.09)

### Tip of the Day

In arm 2, there were 31 revolving tips, and in arm 3, there were 30 revolving tips. Therefore, the probability of a member assigned to the arm 2 seeing a specific tip at least once is .088, and the probability of a member assigned to the arm 3 seeing a specific tip at least once is .091.

A total of 11,431 tips were displayed, of which 564 (4.93%) were clicked on (Table 7). In arm 2, 3622 tips were shown, of which 190 (5.24%) were clicked on. In arm 3, 7809 tips were shown, of which 374 (4.79%) were clicked on. Mixed effect logistic regression revealed no statistically significant differences between the 2 arms regarding the number of tips clicked on (*P*=.25).

**Table 7:** Tips shown and clicked on (N=11,431).

	Arm 2 (n=3622), n (%)	Arm 3 (n=7809), n (%)	Total, n (%)
Tips shown	3622 (100)	7809 (100)	11,431 (100)
Tips clicked on	190 (5.24)	374 (4.79)	564 (4.93)

In arm 2, female members clicked on 171 (5.82%) tips out of the 2937 tips shown to them and male members clicked on 19 (2.8%) tips out of the 682 tips shown to them, whereas in arm 3, females members clicked on 350 (4.94%) out of the 7081 tips shown to them and male members clicked on 24 (3.3%) out of the 722 tips shown to them (Table 8).

There were no statistically significant differences between number of tips clicked on between age groups (Table 9).

Mixed effect logistic regression (Table 10) revealed that female members clicked on more tips than male members (*z* score=2.58; *P*=.01). Although the role of gender and engagement should be more thoroughly examined in future studies, this finding is consistent with other research on platform components [60].

There were no substantial differences between age groups.

**Table 8:** Tips shown and clicked on by gender category (N=10,018).

		Arm 2, n (%)	Arm 3, n (%)	Total, n (%)
**Tips shown to female members**		2937 (100)	7081 (100)	10,018 (100)
	Tips clicked on	171 (5.82)	350 (4.94)	521 (5.20)
**Tips shown to male members**		682 (100)	722 (100)	1404 (100)
	Tips clicked on	19 (2.8)	24 (3.3)	43 (3.06)
**Tips shown to members of other gender identities**		3 (100)	6 (100)	9 (100)
	Tips clicked on	0 (0)	0 (0)	0 (0)

**Table 9:** Tip engagement by age group.

Age group		Arm 2, n (%)	Arm 3, n (%)	Total, n (%)
**Tips shown to members aged 18 to 30 years**		449 (100)	1393 (100)	1842 (100)
	Tips clicked on	26 (5.8)	67 (4.81)	93 (5.05)
**Tips shown to members aged 31 to 40 years**		669 (100)	2130 (100)	2799 (100)
	Tips clicked on	42 (6.3)	110 (5.16)	152 (5.43)
**Tips shown to members aged 41 to 50 years**		677 (100)	1818 (100)	2495 (100)
	Tips clicked on	36 (5.3)	95 (5.23)	131 (5.25)
**Tips shown to members aged 51 to 60 years**		1493 (100)	2260 (100)	3753 (100)
	Tips clicked on	73 (4.89)	91 (4.16)	164 (4.37)
**Tips shown to members aged >60 years**		334 (100)	208 (100)	542 (100)
	Tips clicked on	13 (3.9)	11 (5.3)	24 (4.4)

**Table 10:** Summary table for tip-of-the-day section.

Predictors		OR^a^ (95% CI)	SE	*z* score	*P* value
Arm 3		0.87 (0.69-1.10)	0.1	−1.15	.25
Gender (male members)		0.59 (0.39-0.88)	0.12	−2.58	.01
**Age group (years)**					
	31 to 40	1.08 (0.78-1.49)	0.18	0.44	.66
	41 to 50	0.99 (0.71-1.40)	0.17	−0.03	.98
	51 to 60	0.82 (0.59-1.13)	0.13	−1.22	.22
	>60	1.2 (0.66-2.20)	0.37	0.6	.55

^a^OR: odds ratio.

### To-Do Checklist

As outlined in Textbox 1 and illustrated in Figure 5, the to-do checklist contained 8 items. The members of arm 3 completed an average of 2.7 (34%) out of 8 course components.

The checklist item with the highest engagement was *complete the depression and anxiety test*, which was completed by 51.4% (55/107 of the members; Table 11). The second most popular item was *watch the getting started video*, with 48.2% (923/1914) of the members engaging with the behavioral cue. The checklist item with lowest engagement was *upload my image* with nearly one-fifth (360/1914, 18.8%) of the members engaging with the behavioral cue.

It should be noted that 2 items *read a community post* and *complete the depression and anxiety test* were hidden for some members. This is due to these elements being feature flags and some Evolution Health clients and research partners choosing to hide these features from their membership base.

Although this resulted in a lower number of members seeing these cues and having access to the course components, the percentage of members who viewed the cues and engaged with the components was noteworthy.

Table 12 lists the engagement results for checklist use and gender. Mixed effect logistic regression found that female members clicked on more checklist items than male members (*z* score=2.07; *P*=.04). Although the role of gender and engagement should be more thoroughly examined in future studies, this finding is consistent with other research on platform components [60].

Tables 13 and 14 list the engagement with checklist use and age. Members aged 41 to 50 years and 51 to 60 years were significantly less likely to click on a to-do checklist item (*z* score=2.1; *P*<.04) than members aged 18 to 30 years (*z* score=4.35; *P*<.001). No other differences were found between the age groups.

**Table 11:** To-do checklist item clicked on (N=1914).

To-do checklist item	Clicked, n (%)
Upload my image	360 (18.81)
Complete CBT^a^ session 1	580 (30.3)
Use of the mood tracker or symptom tracker	616 (32.18)
Read a community post	24 (22.4)
Review a worksheet	606 (31.66)
Set goals	605 (31.61)
Complete the depression and anxiety test	55 (51.4)
Watch the getting started video	923 (48.22)

^a^CBT: cognitive behavioral therapy.

**Table 12:** Engagement with the to-do checklist items by gender (N=1914).

To-do checklist item	Women (n=1770, 92.48%), n (%)	Men (n=142, 7.42%), n (%)
Upload my image	338 (19.1)	21 (14.79)
Complete CBT^a^ session 1	549 (31.02)	31 (21.83)
Use the mood tracker or symptom tracker	572 (32.32)	43 (30.28)
Read a community post	20 (29.4)	4 (10.8)
Review a worksheet	572 (32.32)	33 (23.24)
Set goals	566 (31.98)	38 (26.76)
Complete the depression and anxiety test	32 (47.0)	21 (56.8)
Watch the getting started video	862 (48.7)	60 (42.25)

^a^CBT: cognitive behavioral therapy.

**Table 13:** Engagement with the to-do checklist items by age (N=1914).

To-do checklist item	Age group (years; n=1914, 100%), n (%)				
	18 to 30	31 to 40	41 to 50	51 to 60	>60
Upload my image	5 (31.25)	73 (20.74)	100 (20.33)	96 (23.13)	80 (13.65)
Complete CBT^a^ session 1	8 (50)	121 (34.38)	162 (32.93)	147 (35.42)	129 (22.01)
Use the mood tracker or symptom tracker	7 (43.75)	120 (34.09)	163 (33.13)	157 (37.83)	155 (26.45)
Read a community post	1 (50)	11 (30.56)	2 (7.14)	5 (22.73)	4 (26.67)
Review a worksheet	8 (50)	124 (35.23)	158 (32.11)	155 (37.35)	144 (24.57)
Set goals	3 (18.75)	119 (33.81)	158 (32.11)	157 (37.83)	151 (25.77)
Complete the depression and anxiety test	2 (100)	16 (44.44)	13 (46.43)	12 (54.55)	10 (66.67)
Watch the getting started video	9 (56.25)	165 (46.88)	224 (45.53)	244 (58.8)	252 (43)

^a^CBT: cognitive behavioral therapy.

**Table 14:** Summary table for the to-do checklist items.

Predictors		OR^a^ (95% CI)	SE	*z* score	*P* value
Gender (male members)		0.8 (0.65-0.99)	0.08	−2.07	.04
**Age group (years)**					
	31 to 40	0.89 (0.76-1.05)	0.07	−1.4	.02
	41 to 50	0.84 (0.71-0.99)	0.07	−2.1	.04
	51 to 60	0.7 (0.59-0.82)	0.06	−4.35	<.001
	>60	0.73 (0.52-1.03)	0.13	−1.79	.07

^a^OR: odds ratio.

### Comparison of Course Components Completed by Arm

Tables 15 and 16 outline course component completion rates by arm. Using the *set goals* example, 17% (304/1788) of the members in the control arm, 19.8% (171/865) in arm 2, and 31.35% (600/1914) in arm 3 completed the goals exercise.

**Table 15:** Course components completed by arm (N=4567).

Course component	Arm 1 (control; n=1788), n (%)	Arm 2 (RC^a^; n=865), n (%)	Arm 3 (PB^b^, SP^c^, and TD^d^; n=1914), n (%)
Upload my image	13 (0.73)	16 (1.85)	357 (18.65)
Complete CBT^e^ session 1	669 (37.42)	376 (43.47)	806 (42.11)
Use the mood tracker or symptom tracker	366 (20.47)	224 (25.9)	629 (32.86)
Read a community post	46 (39.66)	13 (43.33)	51 (47.66)
Review a worksheet	N/A^f^	21 (2.4)	606 (31.66)
Set goals	304 (17)	171 (19.77)	600 (31.35)
Complete the depression and anxiety test	47 (24.14)	17 (46.67)	60 (51.4)
Watch the getting started video	N/A	24 (2.8)	923 (48.22)

^a^RC: randomized control.

^b^PB: present bias.

^c^SP: social proof.

^d^TD: to-do checklist.

^e^CBT: cognitive behavioral therapy.

^f^N/A: not applicable.

**Table 16:** Summary table for course completion by arm.

Predictors		OR^a^ (95% CI)	SE	*z* score	*P* value
Arm 2		1.31 (1.13-1.52)	0.1	3.59	<.001
Arm 3		1.93 (1.71-2.17)	0.12	10.91	<.001
Gender (male members)		1.05 (0.90-1.23)	0.09	0.6	.55
**Age group (years)**					
	31 to 40	0.97 (0.82-1.15)	0.08	−0.32	.75
	41 to 50	1.12 (0.94-1.33)	0.1	1.26	.21
	51 to 60	0.81 (0.69-0.94)	0.06	−2.7	.007
	>60	0.76 (0.54-1.05)	0.13	−1.66	.10

^a^OR: odds ratio.

The course component with the highest completion rate was *complete the depression and anxiety test,* with 51.4% (60/117) in arm 3, 47% (17/36) in arm 2, and 24.1% (47/195) in arm 1. The course component with the lowest completion rate was *upload my image,* with 18.65% (357/1914) in arm 3, 1.9% (16/865) in arm 2, and 0.73% (13/1788) in arm 1.

As mentioned previously, the 2 items *read a community post* and *complete the depression and anxiety test* were not seen by all members. Some Evolution Health clients do not offer these 2 tools to their membership base. Consequently, there are high engagement percentages but low member use.

For technical reasons, the completion of 2 components *review a worksheet* and *watch the getting started video* were not captured in arm 1. However, about one-third of the members in arm (606/1914, 31.66%) reviewed a worksheet compared to about 2% of those (21/865, 2.4%) in arm 2, and almost half (923/1914, 48.22%) of the members in arm 3 watched *the getting started video* compared to about 3% of those (24/865, 2.8%) in arm 2.

Engagement with 6 of the 8 measurable components was higher in arm 2 than in arm 1 (*z* score=3.59; *P*<.001) and higher in arm 3 than in arm 1 (*z* score=10.91; *P*<.001). Engagement in 8 of the 8 components was higher in arm 3 than in arm 2 (*z* score=4.93; *P*<.001).

Overall, the use of the tips and to-do checklist items resulted in increased engagement. Members in the control arm completed an average of 1.52 course components versus 1.8 course components in arm 2 and 2.11 course components in arm 3.

### CBT Course Completion Rates

The purpose of CBT is to help individuals deal with overwhelming problems; this is achieved by teaching people how to deal with negative thoughts and beliefs [61]. In traditional in-person therapy, the duration of CBT treatment depends on a variety of factors, including health disorders (eg, depression, anxiety, posttraumatic stress disorder, and sleep disorders), symptom severity, duration of therapy, practitioner availability, cost, willingness of the patient to do homework, convenience, culture, treatment settings, and the therapeutic alliance [62].

The recommended duration of CBT varies. Harvard Medical School recommends 30- to 60-minute sessions over 12 to 20 weeks [63], the United Kingdom’s National Health Services recommends 30- to 60-minute sessions once a week or every 2 weeks for 6 to 20 sessions [64], and the Mayo Clinic states that therapeutic encounters can range from 5 to 20 sessions [65].

According to the US National Institutes of Health’s National Library of Medicine, some people undergoing CBT feel much better after a few sessions, whereas others need treatment for several months [66]. In Canada, the Centre for Addiction and Mental Health notes that some people improve in 4 to 6 sessions, whereas others may need >20 sessions [67].

Structured CBT sessions are 1 of the 8 course components in the Evolution Health platform, and like the 7 other course components examined in this study, members’ engagement is optional. The courses Overcoming Depression and Overcoming Anxiety both comprised 9 sessions. On average, 41% (1851/4567) of the members chose to engage with and complete session 1 (Table 15).

**Figure 9:**
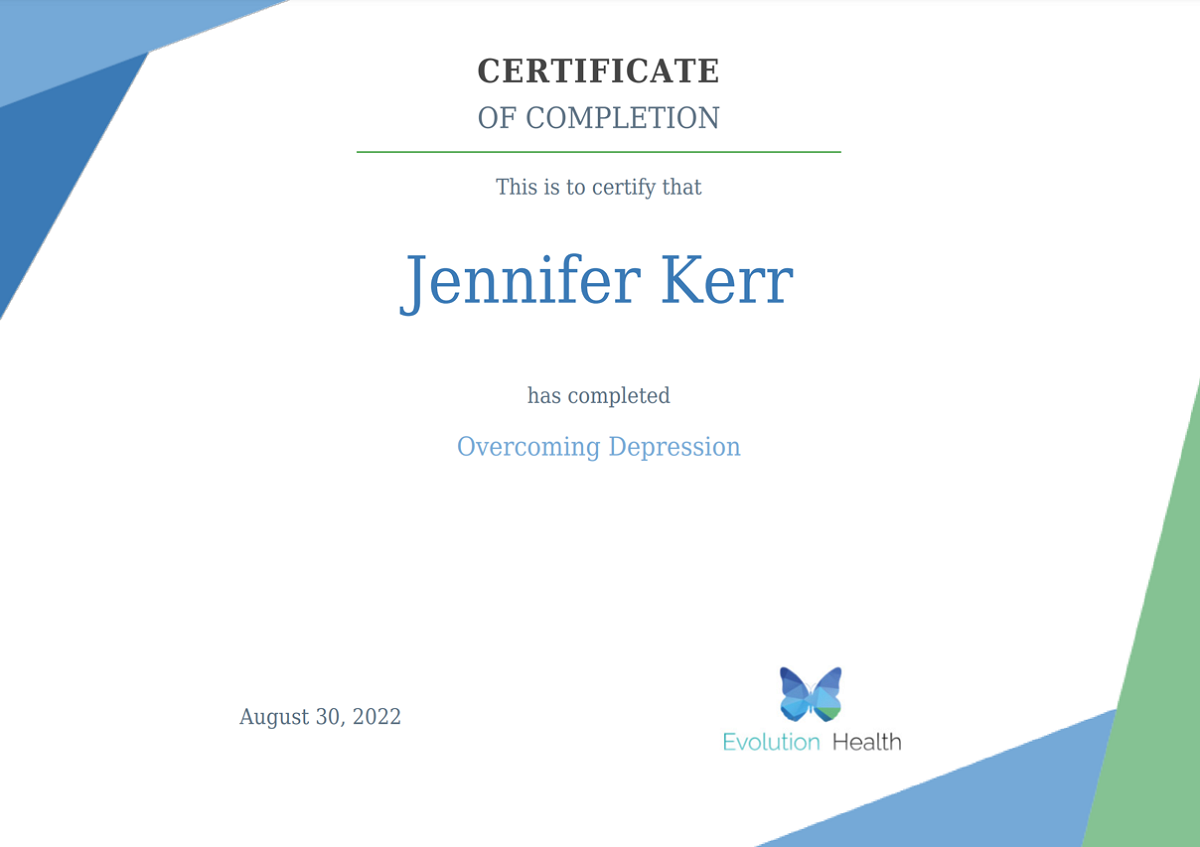
Course completion certificate.

If members completed a course, they received a course completion certificate (Figure 9).

Unlike the platform’s addiction courses, where some members are incentivized by customer sponsors to complete a course (eg, some human resource clients reward their employees for completing the quit smoking behavior change course), to the best of our knowledge, participants in the Overcoming Depression and Overcoming Anxiety courses were not rewarded for course completion or receiving a certificate.

Although the goal of CBT is to decrease symptoms and the duration of treatment varies widely per individual, course completion may be a poor indicator of wellness. However, as engagement is the primary measure of this study and the literature indicates that higher levels of engagement are associated with improved health outcomes, it may be beneficial to form a baseline observation for course completion rates.

Regarding course completion, 31 members in the Overcoming Depression course and 88 members in the Overcoming Anxiety course received course completion certificates. There were no statistically significant differences in the course completion rates between the 3 arms.

In addition, 256 of 4567 members of the N members received a certificate for completing a supplementary 1-session *more help* course, which was less intense and dealt with specific subjects such as overcoming grief, problems in relationships, or role transitions. A greater proportion of members in arm 2 received more certificates for the *more help* course than did the members in arm 1 (*P*=.02). There were no differences of interaction between gender or age groups.

## Discussion

This 3-arm randomized controlled trial tested whether behavioral nudges and prompts could be successfully applied within 2 self-guided treatment digital health programs for anxiety and depression.

### Demographic Characteristics

The average age of the members was 43.8 years, and 87.3% (3987/4567) identified as female members. The average number of log-ins for female members was 2.83 versus 3.89 for male members. The average number of log-ins should be interpreted with caution, as in other studies on the Evolution Health population; engagement appears to follow the properties of power laws [53].

### H1 Summary: Engagement With Behavioral Nudges and Prompts

Our H1 was to analyze whether the members would engage with the tips and to-do checklist.

Regarding gender, mixed effect logistic regression found that female members clicked on more tips than male members (*z* score=2.58; *P*=.01).

Members in arm 3 completed an average of 2.7 (34%) out of the 8 course components featured on the to-do checklist. Engagement ranged from 18.81% (360/1914; *upload my image*) to 51.4% (55/107; *complete the depression and anxiety test*).

Mixed effect logistic regression found that female members clicked on more checklist items than male members (*z* score=2.07; *P*=.04). Members aged 41 to 50 years and 51 to 60 years were significantly less likely to click on a to-do checklist item (*z* score=2.1; *P*=.04) than members aged 18 to 30 years (*z* score=4.35; *P*<.001). No other differences were found between the age groups.

### Secondary Hypothesis Summary: Members’ Preference of Directive Tips in Arm 2 or Social Proof or Present Bias Tips in Arm 3

There were no statistically significant differences in the number of tips clicked on between directive tips in arm 2 and social proof and present bias tips in arm 3 (*P*=.25).

### Tertiary Hypothesis Summary: Tips and to-Do Checklist

Completion rates of 6 out of the 8 course components increased in both experimental arms from baseline use in arm 1. Completion rates in arm 2 were greater than those in arm 1 (*z* score=3.59; *P*<.001), and completion rates in arm 3 were greater than those in arm 1 (*z* score=10.91; *P*<.001).

A total of 2 out of the 8 course tools (*review a worksheet* and *watch the getting started video*) did not have arm 1 baseline statistics. However, completion rates for *review a worksheet* in arm 2 versus arm 3 increased from 2.4% to 31.66%. The completion rate for *watch the getting started video* increased from 2.8% to 48.22%.

Completion rates for the course component *complete CBT session 1* were slightly higher in arm 2 (376/865, 43.5%) than in arm 3 (806/1914, 42.11%). Completion rates of arm 2 were greater than those in arm 1 (*z* score=2.99; *P*=.003), and completion rates in arm 3 were greater than those in arm 1 (*z* score=2.91; *P*=.004). Completion rates of the both arms 2 and 3 were significantly higher than those in arm 1 (669/1788, 37.42%).

The highest increase in course component use was for the *upload my image* component, with 0.73% (13/1788) of the members uploading an image in arm 1, 1.9% (16/865) in arm 2, and 18.65% (357/1914) in arm 3. This is interesting as personalization in digital applications is now common; however, the Evolution Health platform is designed to be anonymous, and before uploading, members were advised to only upload nonidentifying images.

However, high *z* scores require further investigation. For example, high scores may be due to arm 3 members being more likely to complete components than arm 1 members.

### CBT Course Completion Rates

In the literature, efficacy studies analyzing general CBT completion rates are rare, and this is due to complexities surrounding the delivery of CBT for different indications, symptom severity, duration of therapy, practitioner availability, cost, willingness of the patient to do homework, convenience, culture, treatment settings, and therapeutic alliance.

Owing to the nature of the medium, measuring course completion rates in digital health courses is relatively simple. However, digital health courses should not be held to different standards than traditional in-person therapy, where treatment success is often measured by the therapist by observing decreases in symptoms.

In 2005, Eysenbach [11] published *The Law of Attrition*, which recognized that a substantial portion of users drop out of eHealth (digital health) trials before completion and that the high dropout rate makes the efficacy of digital health programs less believable. The paper noted that researchers often compare digital health dropout rates with those of clinical drug trials.

Similar to traditional CBT, where treatment can run from a few weekly sessions to >20 sessions, the digital health literature has shown dose-response effects. Users of self-guided digital health courses interact with devices, not trained therapists. Until digital health platforms can unobtrusively and accurately detect symptom severity, measuring course completion rates will continue to be an inaccurate measure of efficacy.

As higher levels of engagement are associated with improved health outcomes [20,21] and due to the complex methodological issues associated with establishing efficacy rates in population-based digital health CBT programs with high reach, research should continue to focus on strategies that increase adherence and engagement [8-10,12].

### Practical Implications

The arm 3 home page is presently the default setting for the home pages for the Overcoming Anxiety and Overcoming Depression courses. We expect to see increases in course engagement based on the use of behavioral nudges and prompts. Ongoing data collection from all members will contribute to future research.

### Strengths and Limitations

A strength of this study is that it was conducted in an ad libitum environment. Unlike many digital health studies, a large study population in the thousands was leveraged rather than smaller groups. As participants were not aware of participating in the experiment, this limited participant bias and the Hawthorne effect.

A limitation is that, especially due to the anonymity of members, we have no way of identifying participants or validating their demographic information. Although we have no way of knowing whether the registrants are people with depression or anxiety who are seeking help, the removal of nonparticipants should mitigate the effects on the overall results.

Some Evolution Health clients promote certain course tools, or health care professionals may direct their clients to use certain platform attributes. Therefore, the tips or to-do checklist items may not be a factor in their engagement. Second, many members may simply ignore the behavioral cues and complete certain course tools based on their own preference.

The behavioral cues related to *read a community post* and *complete the depression and anxiety test* were not seen by all members. This is due to these elements being feature flags and some Evolution Health clients choosing not to offer these tools to their membership base. Although this resulted in fewer members seeing these cues and having access to the course components, the percentage of members who viewed the cues and engaged with the components was noteworthy.

As this study was designed to form a baseline for future research, there are some methodological issues that can be explored further. For example, unlike the tips presented in arm 2, the use of tips in arm 3 may or may not have been influenced by adding the to-do checklist. Alternatively, using directive tips in arm 3, rather than social proof and present bias tips, may increase the use of the to-do checklist.

Notably, our evidence indicates that nudges and prompts increase engagement in self-guided treatment programs for depression and anxiety. On the basis of these encouraging yet preliminary results, we can proceed with more sophisticated studies that will examine more enhanced strategies designed to increase platform engagement.

### Future Directions

As mentioned earlier, there has been scant published research regarding the implementation of behavioral economic strategies designed to increase engagement in digital health programs. At minimum, this randomized controlled trial was successful in confirming member engagement through the use of these strategies.

As recommended by Aschbacher et al [68] in a recent study involving digital mental health and dose responses, machine learning models can help enable precision by analyzing engagement patterns over time. Combined with another recent paper by Forbes et al [24] analyzing digital interventions for depression, which noted that it is important to develop standardized ways of reporting adherence and engagement so that effective comparisons across different interventions could be measured, baseline outcomes need to be established.

The baseline outcomes of this study can serve as guideposts for future studies. With machine learning models in place and with the goal of improving member outcomes and platform efficacy, Evolution Health considers the following research questions:

- Will members of addiction-focused courses (eg, managing drinking and quitting smoking) follow similar engagement patterns if nudges and prompts are made available to them? Which nudges and prompts can be used universally, and which work best for specific mental health or addiction indications?
- The average member logged into the platform 2.38 times. Can specific tips be introduced at onset to promote log-ins?
- There were no statistically significant differences in the number of tips clicked on between directive tips in arm 2 and social proof and present bias tips in arm 3 (*P*=.25). However, arm 2 featured 31 rotating directive tips and arm 3 featured 15 social proof tips and 15 present bias tips. Which tips were most engaging (eg, directive, social proof, or present bias tips)? Which content areas were most engaging (eg, goal setting, community themed, or specific exercises such as the depression and anxiety tests)? Which tips were the most engaging genders or age groups?
- The average arm 3 member clicked on 2.7 out of 8 to-do checklist items. Which items were these members most likely to click on first? Were there specific patterns of engagement that may influence which tips should be shown to specific members?
- The course components differ in the effort required to complete them. For example, completing a CBT session is more intensive than the few minutes required to complete the depression and anxiety test. Furthermore, one member may take several minutes to contemplate their goals, whereas others may have concrete goals already established. Future research should analyze duration in relation to each course component, as these data may be leveraged to create tailored tips or to-do content for specific user engagement patterns.
- The AVD for members who engaged with depression and anxiety course tools ranged from 6 minutes and 45 seconds, with 7.65 pages viewed, to 24 minutes and 21 seconds, with 12.56 pages viewed. In the future, AVD may be used as a benchmark, as overall engagement may be an important metric to calculate when observing dose-response relationships.
- Members arrived at the platform through the free-to-consumer program and white-label instances licensed by clients that range from employers, insurance companies, employee assistance programs, educational institutions, nonprofit organizations, for-profit health care organizations, and individual therapists. Measuring AVD from these referral sources and patterns of course tool use may assist in creating targeted engagement recommendations.
- The depression and anxiety test has been validated in a separate study, and the algorithm reports whether members qualify for 30 separate mood and anxiety disorders [48]. Symptoms related to these disorders were also collected and reported to the members. Future research should analyze these data to investigate the possible relationships between symptomology, symptom severity, and engagement patterns.
- A recent paper by Forbes et al [24] analyzing patient adherence with digital interventions for depression noted that it is important to standardize reporting adherence and engagement. Our future work will focus on establishing baseline metrics. For example, an acceptable click rate for a tip is x% or an acceptable click rate for a to-do checklist item is y%.
- The platform contains courses for addictive behaviors (eg, smoking cessation and problem drinking), in which members who complete these courses receive a certificate of completion. We are aware that some workforce members received incentives from their human resources department for completing courses and receiving certificates. It may be worthwhile to compare the course completion rates between incentivized addictive behavior courses and nonincentivized mood disorder courses.
- This experiment examined the use of behavioral prompts. Future studies may examine overcoming behavioral barriers such as bounded rationality or choice architecture.

It cannot be assumed that the outcomes observed on the Evolution Health platform will be replicated in other environments. Further research in the combined fields of digital health and behavioral economics is required.

### Conclusions

Members of the Evolution Health platform’s self-guided digital health courses engaged with behavioral nudges and prompts. From this preliminary analysis, it appears that both the tips and to-do checklists increased engagement in course components.

To the best of our knowledge, this is the first randomized controlled trial designed to test the implementation of behavioral nudges and prompts in web-based self-guided courses for mood disorders. The results of this study may be important because efficacy is related to increased engagement.

Owing to its novel approach, the outcomes of this study should be interpreted with caution but may be used as a guideline for future research.

## Appendix

Multimedia Appendix 1 Privacy policy and terms of use.

Multimedia Appendix 2 CONSORT-eHEALTH checklist (V 1.6.1).

## Abbreviations

AVD: average visit duration
CBT: cognitive behavioral therapy
CONSORT-eHEALTH: Consolidated Standards of Reporting Trials of Electronic and Mobile Health Applications and Online Telehealth
H1: primary hypothesis

## Data Availability

The data sets generated during and analyzed during this study are not publicly available due to Evolution Health’s data privacy policy, terms of use, and user agreement but are available from the corresponding author on reasonable request.

Research emerging from this and ongoing data collection will be leveraged to train artificial intelligence models to better understand how to increase and encourage healthy behavior changes. Making these data publicly available may have the opposite effect and enable the development of models that can detect and target susceptible populations.

The rich data set from this study, ongoing engagement data that are continually collected, or other platform data sets can be made available to researchers interested in conducting studies for noncommercial purposes. Interested researchers are encouraged to contact Evolution Health.
