# Supplementary material for: Nudges and Prompts Increase Engagement in Self-Guided Digital Health Treatment for Depression and Anxiety: Results From a 3-Arm Randomized Controlled Trial
Source: JMIR Form Res. 2024 Apr 9;8:e52558. doi: 10.2196/52558 (PMC11040443; doi:10.2196/52558)
Supplement: Multimedia Appendix 1 [file formative_v8i1e52558_app1.pdf]

Evolution Health

https://EvolutionHealth.care

Privacy Policy & Terms of Use

September 2023

This document contains 5 pages

Copyright 2023 by Evolution Health

Terms of Use (https://evolutionhealth.care/site/terms)

Your use of this platform signifies your acceptance of these Terms of Use. We reserve the right, at our sole discretion, to change, modify, delete, or add portions to these Terms of Use at any time. Your continued use of this platform after any such changes constitutes your acceptance of policy enhancements.

1. Disclaimers
   - If you are currently thinking about or planning harm to yourself or someone else, please call 911 or go to the nearest hospital or emergency department.
   - Evolution Health is not a healthcare provider and does not provide medical advice, diagnosis, or treatment. The information on this platform is for educational purposes only.
   - The information, graphics, text, or any materials posted on this platform are intended for educational purposes only, should not be construed as medical advice, and does not replace the advice from your health care provider(s).
   - Access to the platform, and its use, are restricted to personal, non-commercial purposes.
   - We retain the right, in its sole discretion, to make technical improvements or technical upgrades to the platform at any time. This may include the removal of platform elements. No advance notification of upgrades or removals is required.
   - All intellectual property on this platform, including copy, images, official marks, trademarks, or data, are the exclusive property of Evolution Health. Unauthorized use of any such property, without express consent from Evolution Health, is strictly prohibited.

2. Limitation of Liability and Indemnity
   - You agree to defend, indemnify, and hold Evolution Health harmless from any damages arising from your use of this platform.

3. Registration
   - Anyone can view content on this platform. However, use of certain sections require registration. If you choose to register, please become familiar with our Privacy Policy.

4. Community User Agreement
   - The purpose of the community is to provide a place where members can anonymously exchange ideas, opinions, and comments about platform content.
   - Members understand that community content is for the public domain and is not private.
   - All posts and content must specifically address platform content. Any posts that address other topics will be removed and may be subject to the removal of the authors' posting privileges.
   - Members agree not to post any copyrighted material on the platform.
   - Evolution Health, platform owners, managers, employees, interns, and volunteers do not necessary endorse, support, sanction, encourage, verify, or agree with any of the comments, opinion, statements, or posts made within the community.
   - We encourage members to not post any personally identifiable information in the community. Members who post personally identifiable information do so at their own risk.

Copyright 2023 by Evolution Health

- Members may not post or transmit any defamatory, harassing, abusive, mean-spirited, obscene, profane, vulgar, sexually explicit, threatening, or illegal material, or any material containing blatant expression or bigotry, racism, or hate. Evolution Health and/or its employees reserve the right, but is/are not obligated, to edit, review, delete, or refuse to post any material that is submitted to, or is presented on, the platform.
- Members agree not to place any material on the platform that is advertising a product or other service, is advertising another internet site, is invasive of any other's privacy, encourages conduct that would constitute a criminal offense, give rise to civil liberty, or that otherwise violates any local, state, provincial, national, federal, or international law or regulation.
- Members agree to use the platform only for lawful purposes and acknowledge that failure to do so may be subject to civil and criminal liability.
- Members ability to access the platform may be terminated at any time, for any reason, without notice.

5. General
    - These Terms of Use represent the entire understanding with respect to your use of the platform.
    - There Terms of User shall be construed by and enforced in accordance with the laws of the Province of Ontario.
    - These Terms of Use, and all related documents, are available in English. C'est la volonté expresse des parties que la présente convention ainsi que tous les documents qui s'y rattachent soient rédigés en anglais.

Copyright 2023 by Evolution Health

Privacy Policy (https://evolutionhealth.care/site/privacy)

This platform provides customized tools and information that help users recognize and manage symptoms related to addictions and mental health. If you use this platform you will be asked for some anonymous personal information. As you proceed through the program you will be asked for other information specific to your progress.

The following outlines the type of information we collect, how it is used, and describes our Privacy Policy regarding the use of this data.

What Kind of Information Is Collected and Why?

- **Gender:** some materials are gender specific and are based on success strategy research.
- **Age:** some of the feedback we provide you is based on how your age relates to the Cognitive Behavioral Therapy (CBT).
- **First name:** this program personalizes your answers and creates a program that is specific to you. Please do not use your last name. To protect your anonymity, we suggest that you use a nickname or an alias.
- **Email ID:** Your Email ID are only used as part of your sign-in process, or to send you community updates if you select that option. We do not sell email addresses. Because email addresses may contain information that can potentially identify you we suggest that you use a free, anonymous address.
- **Data Specific to Your Mood and Personal Progress:** This data is only used to provide you with personal feedback and customized plans.

We do not ever sell or share email addresses, names, or addresses to third parties and we will not contact you with any marketing offers or spam.

How Is This Data Used?

- This data will be used to create your personal program.
- Your data will be grouped with other people's data so we can understand how to improve the platform for other users.
- General, anonymous information will be analyzed to determine the effectiveness of our platform for culturally diverse and international-based group of individuals.
- Your data will be used to collect anonymous efficacy and clinical data that is used in validating the effectiveness of this software platform by professional researchers.
- We have never, nor will be ever, sell email identification, names, or addresses with third parties.

Your Involvement in a World-Wide Treatment Community

This platform is unique in that it allows you to easily and anonymously become an active member of a worldwide, ongoing CBT program.

This platform has continually evolved since its first launch in 2000. Research data gathered on the tens of thousands of members has only been reviewed on the basis of population and has

Copyright 2023 by Evolution Health

only been scientifically analyzed by Evolution Health Systems and its advisors. No personal data has been, or will ever be, sold.

Your personal involvement in a world-wide community overcoming mood disorders will help future generations as we continually upgrade and improve this program and publish outcome data in peer-reviewed academic journals.

Your opinion and support are valuable to us. Please contact us if you have any questions or concerns

Copyright 2023 by Evolution Health
